# Optimal control of quantum state preparation and entanglement creation in two-qubit quantum system with bounded amplitude

**DOI:** 10.1038/s41598-023-41688-z

**Published:** 2023-09-07

**Authors:** Xikun Li

**Affiliations:** 1 School of Physics and Optoelectronic Engineering, Anhui University, Hefei, 230601 China; 2 Max-Planck-Institut für Physik komplexer Systeme, 01187 Dresden, Germany; 3 Department of Physics and Astronomy, Aarhus University, 8000 Aarhus, Denmark

**Keywords:** Quantum information, Qubits

## Abstract

We consider the optimal control problem in a two-qubit system with bounded amplitude. Two cases are studied: quantum state preparation and entanglement creation. Cost functions, fidelity and concurrence, are optimized over bang-off controls for various values of the total duration, respectively. For quantum state preparation problem, three critical time points are determined accurately, and optimal controls are estimated. A better estimation of the quantum speed limit is obtained, so is the time-optimal control. For entanglement creation problem, two critical time points are determined, one of them is the minimal time to achieve maximal entanglement (unit concurrence) starting from the product state. In addition, the comparisons between bang-off and chopped random basis (CRAB) are made.

## Introduction

Quantum optimal control (QOC) is crucial to quantum information processing tasks, such as quantum computation and quantum communication. In these tasks, complex quantum systems are engineered and manipulated, e.g. to achieve target quantum gates and target quantum states^1–4^. In certain cases, the adiabatic operations, which are generally executed very slowly, are desired in experiments, because we wish to avoid heating the sample and to guarantee the target gate/state is prepared with perfect fidelity^5^. However, in experiments the decoherence and noise from the environment often make such slow operations impossible. Therefore, speedup the time evolution by applying fast and robust controls is sensible^6,7^.

Quantum optimal control theory, which is proposed to solve the problems mentioned above, has been widely applied in various physical systems such as NMR^8^, Bose-Einstein condensate^9^, cold atoms in optical lattices^10,11^. One of the important topics in QOC theory is to search the time-optimal control with which the transitions are finished in the *minimal* time. In the context of QOC, the minimal time is generally called the quantum speed limit (QSL)^12^. And the temporal shape of the corresponding control field is called time-optimal control. Analytic solutions are available for several cases where the quantum systems considered are in low-dimensional^13–23^. For multiple-level quantum systems where analytical results are absent, one has to perform numerical optimization.

Roughly speaking, we rely on two classes of optimization: local optimization algorithms, like Krotov^24^, GRAPE^8^, CRAB^25^, GROUP^26^ and GOAT^27^, as well as global ones such as differential evolution (DE) and covariance matrix adaptation evolution strategy (CMA-ES)^10,28^. Machine learning techniques, especially reinforcement learning is another promising method^29^.

The time-optimal problem of two-qubit system with unbounded amplitude is studied in Ref.^30^. However, in real experiments the range of tuning parameters of apparatus is finite, thus constraints in general exist on the control field, e.g., the amplitude is bounded. In such cases the appearance of local suboptimal traps in the quantum control landscape makes the QOC problem nontrivial^31,32^.

In this paper we consider the optimal problem in a two-qubit system with bounded amplitude. We study two problems: quantum state preparation and entanglement creation. For the first one, one wants to achieve the target quantum state with QSL, and to find the temporal shape of time-optimal control. For the latter, we are interested in the problem that for given total duration, how large the maximal entanglement can be obtained^33–38^.

The quantum state preparation problem in two-qubit quantum system was investigated from the viewpoint of quantum control phase transition in Ref.^39^. Numerically the bang-bang protocol is optimized using stochastic descent (SD) method to approximate the optimal control fields and to estimate QSL. Three critical time points $T_c$, $T_{\textrm{sb}}$ and $T_{\textrm{QSL}}$ are estimated by studying the behavior of several physical quantities, e.g., correlation. Moreover, the optimal controls in different phase regions are approximated by averaging the optimized bang-bang controls. However, the values of these critical time points are not accurate, and the optimal controls estimated behave like bang-off control, rather than bang-bang.

We wish to estimate the values of critical points and the temporal shape of optimal controls more accurately using the bang-off control proposed in Ref.^40^. Employing the same scenario, we optimize over the bang-off controls to estimate the optimal control for the problem of quantum state preparation and entanglement creation. In addition, we compare our method with one of the state-of-the-art methods, chopped random basis (CRAB).

## Model

We consider the symmetrically coupled two-qubit Hamiltonian studied in Ref.^39^, which is described in the following:

1 $$\begin{aligned} H(t) =-2g S_1^z S_2^z -h_z(S_1^z+S_2^z)-h_x(t) (S_1^x+S_2^x) \end{aligned}$$

where $g=h_z=1$ are the interaction strength and static magnetic field along the *z* direction, and $h_x(t)$ is the time-dependent control field along the *x* direction. $S_1^z=\sigma _z/2$ is spin-1/2 Pauli operators for the first qubit. The bounded control field $h_x(t)$ is a real function under constraint $|h_x(t)|\le M$. The dynamics of the system is governed by the Hamiltonian $\textrm{d} |\psi (t) \rangle / \textrm{d}t = -\textrm{i} H(t) |\psi (t) \rangle$, where we set $\hbar =1$, starting from the initial state $|\psi _{i}\rangle$.

For the quantum state preparation problem, we set the cost function to be the fidelity *F* defined as follows:

2 $$\begin{aligned} F(h_x(t),T)&=|\langle \psi _{t}|\mathcal {T}\exp (- \textrm{i}\int _0^T H(t) \textrm{d}t |\psi _{i}\rangle |^2 \nonumber \\&=|\langle \psi _{t}|\psi _{f}\rangle |^2. \end{aligned}$$

where $\mathcal {T}$ is the time-ordering operator. *T* is the total duration of time evolution, and $|\psi _{f}\rangle$ is the final state. The initial state $|\psi _{i}\rangle$ is prepared in the ground state of Hamiltonian (1) with $h_x=-2$, and the target state $|\psi _{t}\rangle$ is set to be the ground state of Hamiltonian with $h_x=2$.

For the entanglement creation problem, we use concurrence to measure the entanglement of two-qubit pure state. A general two-qubit pure state can be expressed as $| \psi \rangle = a |00\rangle + b |01\rangle + c |10\rangle + d |11\rangle$, where *a*, *b*, *c*, *d* are complex numbers with normalization condition $|a|^2+|b|^2+|c|^2+|d|^2=1$. The concurrence of two-qubit pure state is defined in the following^41^:

3 $$\begin{aligned} C(| \psi \rangle ) = 2|ad-bc|. \end{aligned}$$

Specially, the final state of the two-qubit, which follows the Schrödinger evolution with control field $h_x(t)$, is denoted as $| \psi _f (T) \rangle = a(T) |00\rangle + b(T) |01\rangle + c(T) |10\rangle + d(T) |11\rangle$. Thus the concurrence of final state is $C(T)= 2|a(T)d(T)-b(T)c(T)|$.

For quantum state preparation problem (entanglement creation problem), we want to find the control field $h_x(t)$ which maximizes the fidelity $F(h_x(t),T)$ ($C((h_x(t),T)$) for given *T*. We refer to such $h_x(t)$ as the *optimal control* for *T*. Particularly, for quantum state preparation problem, we wish to estimate the quantum speed limit $T_{\textrm{QSL}}$ with which the target state is obtained with unit fidelity $F=1$. For entanglement creation problem, we want to calculate the minimal time $\tau _{\textrm{min}}$ such that the unit concurrence $C=1$ is reached. Notice that different from the quantum state preparation problem, the number of two-qubit pure states with unit concurrence is *infinite*, while there is, in general, only *one* target state for quantum state preparation problem.

Employing the same scenario in Ref.^40^, we optimize *F* and *C* over bang-off control. The bang-off control refers to a finite concatenation of *bang* controls *P* and *N*, and *off* control 0. *P* (*N*) is short for Positive (Negative) where $h_x(t) = M$ ($h_x(t) = -M$) and 0 is $h_x(t) = 0$. The control field is represented by the type—a sequence of *P*, *N* and 0—and vector of durations ${\textbf{t}}=[t_1,t_2,...]$. For example, the control field $P_{t_1} 0_{t_2} N_{t_3}$ is defined in the following

4 $$\begin{aligned} h_x(t)= \Bigg \{ \begin{matrix} M&{} 0 \;\;\le t< t_1 \\ 0 &{} \;\; t_1\le t < t_1+t_2 \\ -M &{} \;\; t_1+t_2\le t \le t_1+t_2+t_3, \\ \end{matrix} \end{aligned}$$

where the order of letter sequence is from left to right. For the example above, the switch number is two $N_s=2$, and the bang-off control is of type *P*0*N* which is switched from bang (*P*) to off (0), then to bang (*N*). The number of possible types $N_{\textrm{type}}$ is at most $3\times 2^{N_s}$ for a given number of switches $N_s$. One possible limitation of bang-off method is that the number of possible types grows exponentially fast as $N_s$ increase. Thus the optimization is resource consuming. However, for certain initial/target quantum states, $N_{\textrm{type}}$ can be further reduced. Here we take $M=4$ such that $|h_x(t) \le 4|$.

For given *T*, we optimize *F* (and *C*) starting from $N_s=0$. For each type with given $N_s$ we optimize the vector of durations ${\textbf{t}}$ using quasi-Newton method. Quasi-Newton method is a gradient-based method. In addition, the Hessian matrix is estimated such that the computation is not costly. To be specific, we employ the BFGS method to optimize the bang-off control.

We denote $F_i$ the maximal fidelity obtained using control fields with $N_s=i$, and the difference of maximal fidelity $\Delta F_{i} \equiv F_{i+1}-F_{i}$. Similar notations are defined for *C*. Once $\Delta F$ ($\Delta C$) is zero or vanishing small as $N_s$ increases, we stop the optimization and estimate the optimal control with the corresponding optimized control field.

## Quantum state preparation

For the quantum state preparation problem of two-qubit system, it is helpful to imagine the system as an interacting two spin system. These two spins are controlled via static magnetic field, and we wish to transfer from the ground state of system with initial value of magnetic field to the ground state of the system with another value.

**Figure 1:**
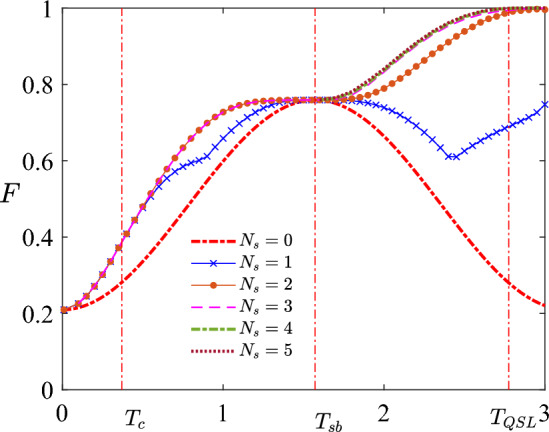
Maximal fidelity *F* as a function of the total duration *T* obtained with different number of switches from $N_s=0$ to $N_s=5$. Three critical time points are $T_c = 0.37037$, $T_{\textrm{sb}}=\pi /2$, $T_{\textrm{QSL}}\approx 2.775$. For $T \in [0,T_c]$, the optimal control field is $P_{T/2}N_{T/2}$. For $T \in (T_c,T_{\textrm{sb}})$, the optimal control field is $P_{t_1}0_{T-2 t_1}N_{t_1}$. For $T=T_{\textrm{sb}}$, the optimal control is $0_{\pi /2}$. For $T \in (T_{\textrm{sb}},T_{\textrm{QSL}}]$, the best *F* increases as $N_s$ increases. When $N_s\ge 6$, however, the increment of *F* is too little, thus are not shown in this figure.

By observing the behavior of $\Delta F_i$ we estimate the values of three critical time points. The values of critical time points obtained here are more accurate than those obtained in Ref.^39^. They are $T_c=0.37037$, $T_{\textrm{sb}}=\pi /2$, and $T_{\textrm{QSL}}\approx 2.775$; cf. Fig. 1. For $T \in (0,T_c]$, the system is in the *overconstrained* phase. In this region, the control landscape is very easy, i.e., there is only one minimum, which is global, such that it is very easy to find the optimal control. For the *correlated and glassy* phase ($T \in (T_c,T_{\textrm{sb}}]$), the optimal control is still symmetric, i.e., $h_x(t)=-h_x(T-t)$. For the *symmetry-broken* phase ($T > T_{\textrm{sb}}$), the optimal control fields are not symmetric anymore. Refer to Ref.^39^ more for details.

In addition, we conjecture that the optimal controls in overconstrained phase ($T \in (0,T_c]$) and correlated and glassy phase ($T \in (T_c,T_{\textrm{sb}}]$) are bang-off controls. This might suggest that for $T \le T_{\textrm{sb}}$ the optimal control over the singular regions (where control does not take value of *M* or $-M$) takes zero value, i.e., the singular control is the off control $h_x(t)=0$. For the symmetry-broken phase ($T \in (T_{\textrm{sb}}, T_{\textrm{QSL}})$), however, the numerical results suggest that the singular control is not off control anymore, but takes certain non-zero value which is between the upper and lower boundary. It means that the optimal control field is not bang-off anymore.

We find that the optimal types found with different $N_s$ is of type *P*...*N*. This results from the fact that the initial state is the ground state of Hamiltonian with negative value $h_x=-2$, while the target state is that of positive value $h_x=+2$. The details are in the following.

### Optimal control for $T \in (0,T_c]$

**Figure 2:**
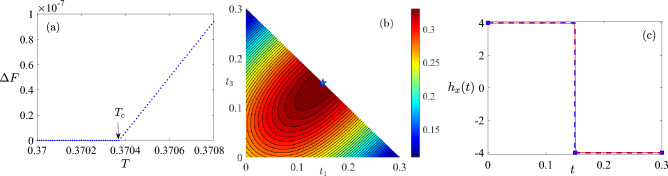
The optimal control is $P_{T/2}N_{T/2}$ for $T \in [0,T_c]$. (**a**) $\Delta F = F_{2}- F_{1}$ versus the total duration *T*. $F_{N_s}$ is the best fidelity obtained with number of switch $N_s$. $\Delta F=0$ when $T\le T_c$. However, $\Delta F>0$ when $T>T_c$. $T_c=0.37037$ is indicated by an arrow. (**b**) The quantum control landscape of fidelity as a function of $[t_1,t_3]$ for $N_s=2$ control $P_{t_1}0_{t_2}N_{t_3}$ with $t_1+t_2+t_3=0.3$. The maximal fidelity is indicated by a blue pentagram whose location is $[t_1,t_3]=[0.15,0.15]$, which means $t_2=0$ and the $N_s=2$ control is reduced to $N_s=1$ control $P_{0.15}N_{0.15}$. (**c**) The optimal control (blue dashed), which is bang-bang, and the optimized control with best fidelity found by CRAB (red solid) for $T=0.3$.

For $T \in (0,T_c]$, the optimal control protocol is $P_{T/2}N_{T/2}$ in the overconstrained region. The best fidelity obtained with $N_s >1$ is equal to that with $P_{T/2}N_{T/2}$ for $T \in (0,T_c]$. In addition, the optimal control fields obtained with $N_s>1$ is $P_{T/2}N_{T/2}$.

In Fig. 2a we show $\Delta F=F_2-F_1$, which is the difference between the best fidelity obtained with $N_s=2$ and that with $N_s=1$. It is observed that $\Delta F=0$ for $T \le 0.37037$, whereas $\Delta F>0$ for $T > 0.37037$. The same result holds for $N_s\ge 3$. In such way we locate the value of $T_c=0.37037$ which is more accurate than the one obtained in^39^ where $T_c$ is approximately equal to 0.38.

In Fig. 2b we show the quantum control landscape over the control field of type *P*0*N* with $T=0.3$. The maximal fidelity is obtained with control $P_{T/2}0_0 N_{T/2}$, which is in fact $P_{T/2} N_{T/2}$ with $N_s=1$. The same conclusion is true for all $T \in (0,T_c]$. In Fig. 2c we further demonstrate the optimized control field using CRAB method. We observe that the temporal shape of the optimized control field using CRAB method deviates vanishingly small from that of bang-bang control, so does the fidelity obtained using two methods.

### Optimal control for $T \in (T_c,T_{\textrm{sb}}]$

**Figure 3:**
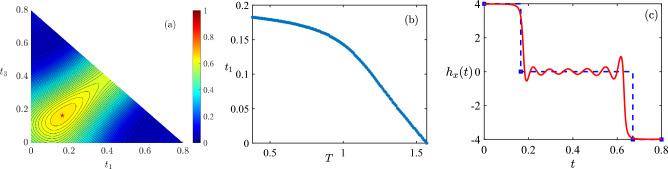
The optimal control is of type $P_{t_1}0_{T-2t_1}N_{t_1}$ for $T \in (T_c,T_{\textrm{sb}}]$. (**a**) The quantum control landscape of fidelity as a function of $[t_1,t_3]$ for $N_s=2$ control $P_{t_1}0_{t_2}N_{t_3}$ with $t_1+t_2+t_3=0.8$. The maximal fidelity is indicated by a red pentagram whose location is $[t_1,t_3]=[0.1648,0.1648]$. (**b**) $t_1$ as a function of the total duration *T* for the optimal control $P_{t_1}0_{T-2t_1}N_{t_1}$. $t_1=0$ when $T=\pi /2$, thus the optimal control is $0_{\pi /2}$. (**c**) The optimal control $P_{t_1}0_{T-2t_1}N_{t_1}$ with $t_1=0.1648$ (blue danshed), which is bang-off, and the optimized control with best fidelity found by CRAB (red solid) for $T=0.8$.

For $T \in (T_c,T_{\textrm{sb}}]$, the optimal control protocol is of $N_s=2$ type $P_{t_1}0_{t_2}N_{t_1}$ with $2 t_1 + t_2=T$. In addition, the optimal control fields obtained with $N_s \ge 3$ reduce to the control of type $P_{t_1}0_{t_2}N_{t_1}$.

For $N_s=2$, we have numerically checked that the optimal control is of type *P*0*N* within all 12 types when $T \in (T_c,T_{\textrm{sb}}]$. Moreover, the best fidelity is obtained with a special control field $P_{t_1}0_{T-2t_1}N_{t_1}$, i.e., the first duration being equal to the last one $t_3=t_1$. In Fig. 3a it is shown that the quantum control landscape of control field $P_{t_1}0_{T-t_1-t_3}N_{t_3}$ with $T=0.8$. The maximal fidelity is obtained with $t_1=t_3=0.1648$. Similar results hold for $T \in (T_c,T_{\textrm{sb}}]$. Therefore, the optimal duration vector is $[t_1,T-2t_1,t_1]$. The value of $t_1$, shown in Fig. 3b, is determined numerically. Notice that $t_1=0$ when $T=T_{\textrm{sb}}$, thus the optimal control field reduces to $0_{\pi /2}$.

In Fig. 3c we demonstrate the optimized control field obtained using bang-off control and the one using CRAB method for $T=0.8$. The fidelity obtained with latter is a little smaller than the former. For the optimized control using CRAB, the oscillation around $h_x(t)=0$ serves as a reasonable approximation to the off control. In addition, in Ref.^39^ the temporal shape of optimal control for $T \le T_{\textrm{sb}}$ is obtained by averaging the optimized bang-bang controls, which turns out to be approximately bang-off. It is also worth noting that in a two-level system with bounded amplitude, the time-optimal control for certain target states is indeed bang-off type^18^. Considering the above results, we conjecture that the optimal controls for $T \in (0,T_{\textrm{sb}}]$ are bang-off controls.

### Optimal control for $T \in (T_{\textrm{sb}}, T_{\textrm{QSL}}]$

**Figure 4:**
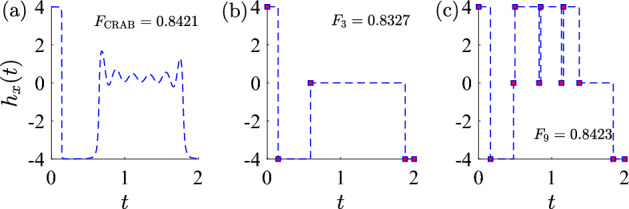
The optimized control fields found with different types for $T=2 \in (T_{\textrm{sb}},T_{T_{\textrm{QSL}}})$. (**a**) The optimized control field found using CRAB with the best fidelity obtained. (**b**) One of the optimized control fields found with $N_s=3$ bang-off control, and the best fidelity obtained is smaller than the value obtained using CRAB. (**c**) One of the optimized control fields found with $N_s=9$ bang-off control, and the best fidelity obtained is larger than the value obtained using CRAB.

In the symmetry-broken phase region $T \in (T_{\textrm{sb}}, T_{\textrm{QSL}})$, the double degeneracy of optimal control field is displayed by two optimal control fields with the relation $h_1^{\textrm{opt}}(t)=-h_2^{\textrm{opt}}(T-t)$^39^. Here we show the results for one of the optimal controls.

**Figure 5:**
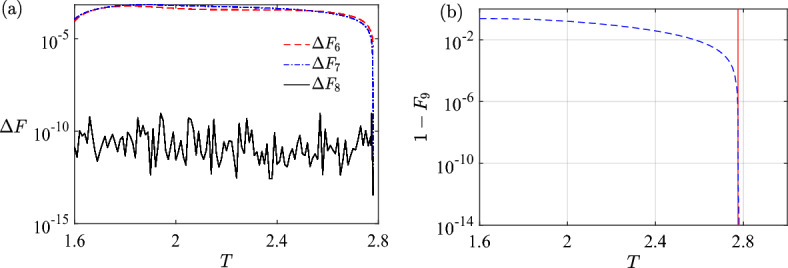
(**a**) The differences of best fidelity $\Delta F$ obtained with switch number from $N_s=6$ to $N_s=9$ as a function of total duration *T*, where $\Delta F_6 = F_7-F_6$ and so on. (**b**) The infidelity $1-F$ as a function of total duration *T* over control with $N_s=9$ (blue dashed line). The estimation of quantum speed limit is marked by a vertical asymptotic line, where two vertical lines indicate the estimation of quantum speed limit obtained by GRAPE $T\approx 2.775$ (red solid line) and by symmetric ansatz $T\approx 2.907$ obtained in Ref.^39^, respectively.

Different from the case $T<T_{\textrm{sb}}$, the optimal control with $T \in (T_{\textrm{sb}}, T_{\textrm{QSL}})$ is not bang-off anymore. The first evidence is the behavior of $F_{N_s}$ as $N_s$ increases. For the case $T \le T_{\textrm{sb}}$, the maximal fidelity $F_{N_s}$ stops increasing for small $N_s$ ($N_s=1$ for $T \le T_c$ and $N_s=2$ for $T_c \le T \le T_{\textrm{sb}}$). In contrast, $F_{N_s}$ grows as $N_s$ increases when $T >T_{\textrm{sb}}$; cf. Figs. 1 and 5a. Such a large number of switches might approximate a smooth function over the singular region.

The second evidence is that the best fidelity obtained using bang-control field with $N_s \le 8$ is smaller than the one with CRAB; cf. Fig. 4. It is interesting to note that the optimized control using CRAB and the one with $N_s=3$ bang-off control approximate to each other very well. Given the fact that the bang-off control with small $N_s$ in general performs worse than CRAB, it might suggest that the optimal control field over the singular region takes a smooth structure (not necessarily to be CRAB), rather than bang-off which is piece-wise constant. In addition, in Fig. 4 all three optimized control field has bang control at the beginning and end. A similar result is reported in Ref.^42^. Thus, the optimal control might has a structure of “bang-annealing-bang”^42^.

We emphasize that even if the optimal control for $T \in (T_{\textrm{sb}}, T_{\textrm{QSL}}]$ might have a bang-annealing-bang structure, we can still approximate the optimal control with the bang-off control whose temporal shape is simple. In the following,we estimate the quantum speed limit and the time-optimal control using the bang-off control.

In Ref.^39^ the quantum speed limit is estimated using GRAPE $T_{\textrm{GRAPE}} \approx 2.775$, and by optimizing the three-pulse symmetric anstaz, which is in fact $N_s=6$ bang-off control of one special type $h_x(t)=P_{t_1}0_{t_2}P_{t_3}0_{t_4}N_{t_3}0_{t_2}N_{t_1}$ with $h_x(t)=-h_x(T-t)$. The estimation of quantum speed limit obtained using this anstaz is $T \approx 2.907$. In fact, the estimation of $T_{\textrm{QSL}}$ can be better using the generic unsymmetrical bang-off control. From Fig. 5b we observe that the estimation of $T_{\textrm{QSL}}$ using $N_s=4$ bang-off controls is better than the anstaz.

In Fig. 5b we show the infidelity $1-F$ as a function of *T* obtained using the bang-off control. The optimal control protocol with $N_s=9$ is of type *P*0*N*0*NP*0*P*0*N*. The unit fidelity is reached $F=1-\mathcal {O} (10^{-15})$ with two optimal duration vectors: ${\textbf{t}}^*= [0.232,0.244,0.561,0.317,0.017,0.093,0.858,0.044,0.241, 0.173]$, and another one which is the flipped vector of ${\textbf{t}}^*$. The estimation of QSL is $T_{\textrm{QSL}}\approx 2.775$ by using bang-off control with $N_s=9$. This is approximately equal to the one obtained with GRAPE, and less than $T=2.907$ which is obtained in Ref.^39^. See Fig. 5b for illustration. While the control fields obtained using GRAPE are continuous, the temporal shape of bang-off control is simpler than the former.

## Entanglement creation

**Figure 6:**
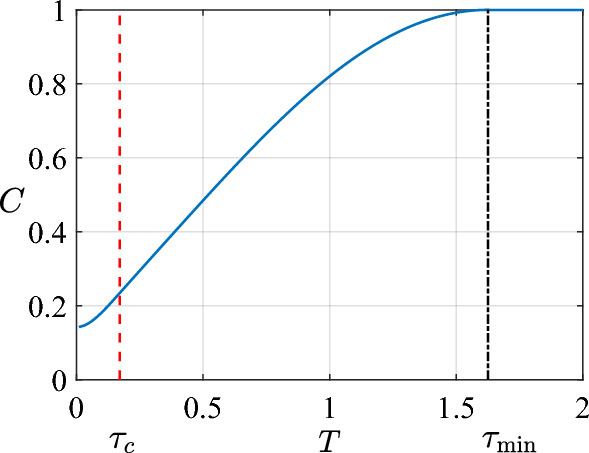
Concurrence C of the optimal control as a function of total duration *T*. Two critical time points are $\tau _c \approx 0.380$, and $\tau _{\textrm{min}} \approx 1.779$.

In this section we estimate the optimal control for the problem of entanglement creation. The system is driven by the Hamiltonian (1) with the initial state $|00\rangle$. For given *T*, we wish to maximize *C* over the bang-off control field, and obtain the optimal control field. Same as the quantum state preparation problem, the quantum system describe with Eq. 1 can be considered as the interacting two spin system. The entanglement is created by the interacting between two spins. We start from a separable state with no entanglement, and wish to find the minimal time to reach unit concurrence and the corresponding time-optimal control, by optimizing the static magnetic field. Similar to the quantum state preparation problem, two critical time points are found: $\tau _c \approx 0.380$, and $\tau _{\textrm{min}} \approx 1.779$. See Fig. 6 for illustration.

### Optimal control for $T \in [0,\tau _c]$

**Figure 7:**
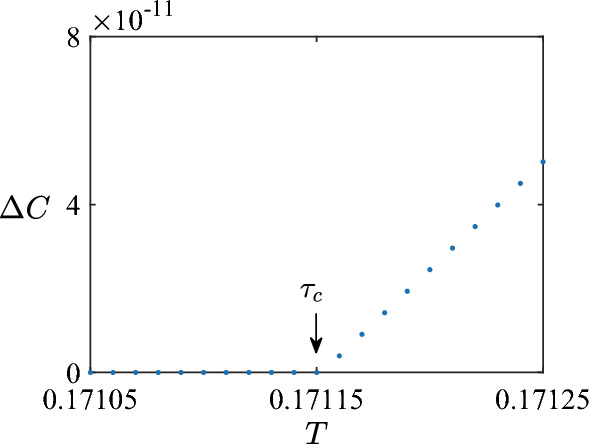
$\Delta C_0 \equiv C_{1}- C_{0}$ versus the total duration *T*. The critical time point is $\tau _c=0.380181$. $\Delta C_0=0$ when $T\le \tau _c$, and $\Delta C_0 >0$ when $T > \tau _c$. For $T \in [0,\tau _c]$, the optimal control field is $P_T$ and $N_T$.

For $T \in [0,\tau _c]$ the optimal control is $P_T$ and $N_T$. This is numerically confirmed by two steps. First, we calculate $\Delta C_i \equiv C_{i+1} - C_i$, where $C_i$ is the maximal concurrence obtained with $N_s=i$, for various values of switch number. All these $\Delta C_i$ are zero when $T \le 0.380181$. However, $C_1 > C_0$ when $T > 0.380181$. Second, when $T \in [0,\tau _c]$, we find that $C_0$ is obtained with the control field $P_T$ and $N_T$, and same for $C_i$ ($i \ge 1$). Therefore, the first critical time point is $\tau _c = 0.380181$, and the optimal control with $T \in [0,\tau _c]$ is $P_T$ (and $N_T$) .

In Fig. 7 we show $\Delta C_0$ as a function of *T* for example. Following the terminology in Ref.^39^, we call the region with $T \in [0,\tau _c]$ the overconstrained phase, because the search for global minimum is easy.

### Optimal control for $T \in (\tau _c, \tau _{\textrm{min}}]$

We have numerically calculate the concurrence *C* with different values of $N_s$ for $T>\tau _c$. For given *T*, the best value of *C* stops increasing when $N_s$ is larger than three. In addition, the control fields optimized with $N_s=4$ are reduced to the ones with $N_s=3$. Therefore, we conjecture that the optimal control is of type $N_s=3$ for $T>\tau _c$.

**Figure 8:**
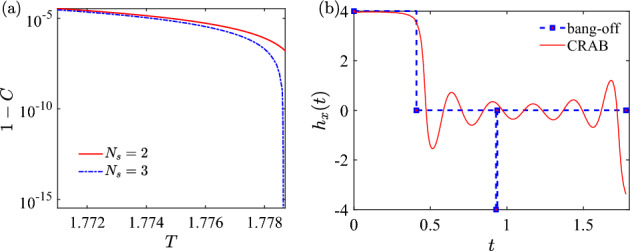
(**a**) Inconcurrence $1-C$ as a function of total duration *T* for $N_s=2$ and $N_s=3$ with the initial state $|00\rangle$. (**b**) The unit concurrence is obtained by the optimal control field $P_{t_1}0_{t_2}N_{t_3}0_{t_4}$. In addition, the optimized control is obtained using CRAB for $\tau _{\textrm{min}} \approx 1.779$. See main text.

In Fig. 8a we show the inconcurrence $1-C$ as a function of *T* for number of switches $N_s=2$ and $N_s=3$. The unit concurrence is reached with $T \approx 1.779$. This is also indicated by a vertical asymptote in the logarithmic inconcurrence $1-C$. See Fig. 8a for illustration. Therefore, we estimate the minimal time to reach unit concurrence being $\tau _{\textrm{min}} \approx 1.779$. One of the time-optimal control is $P_{t_1}0_{t_2}N_{t_3}0_{t_4}$ with the time vector being ${\textbf{t}}=[0.4086, 0.520, 8.138\times 10^{-3}, 0.841]$. As a comparison, we employ the CRAB method to optimize the control field with $\tau _{\textrm{min}}$, and the minimal inconcurrence obtained is of order $10^{-6}$, which is ten orders of magnitude greater than the one obtained with our method; cf. Fig. 8b.

**Figure 9:**
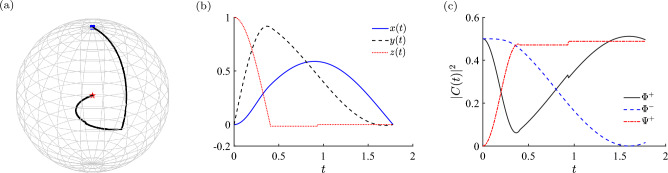
The optimal trajectory obtained using the optimal control shown in Fig. 8b. (**a**) The trajectory of reduced density matrix obtained using the optimal control at the Bloch sphere by tracing one qubit. The initial state on the north pole is marked by the blue square, and the final state which is on the centre is marked by a red pentagram. The optimal trajectory is shown in black solid line. (**b**) The corresponding Cartesian coordinate shown in (**a**). (**c**) The coefficients in Eq. (5) with respect to three Bell states which span the triplet subspace of two-qubit Hilbert space.

In Fig. 9a we show the trajectory of reduced density matrix on the Bloch sphere by tracing one qubit. The initial state is indicated by the blue square, and the final state in the centre of Bloch sphere is indicated by a red pentagram, which means the final state of two-qubit state is one of maximally entangled two-qubit state, i.e. $C=1$. In Fig. 9b we further show the Cartesian coordinate [*x*(*t*), *y*(*t*), *z*(*t*)] of the optimal trajectory on the Bloch sphere.

Furthermore, we investigate the optimal trajectory in the full two-qubit picture. Notice that the initial state $|00\rangle$ is inside the Hilbert subspace of triplet manifold, and that the Hamiltonian (1) is invariant by exchanging the two qubits^39^. Therefore, the time evolution of two-qubit system is inside the Hilbert subspace of triplet manifold. The time evolving state is the superposition of three Bell states, i.e., $|\Phi ^+\rangle =(|00\rangle +|11\rangle )/\sqrt{2}$,$|\Phi ^-\rangle =(|00\rangle -|11\rangle )/\sqrt{2}$, and $|\Psi ^+\rangle =(|01\rangle +|10\rangle )/\sqrt{2}$. We monitor the three coefficients of three Bell states

5 $$\begin{aligned} |\psi (t)\rangle = C_1(t)|\Phi ^+\rangle + C_2(t)|\Phi ^-\rangle + C_3(t)|\Psi ^+\rangle . \end{aligned}$$

In Fig. 9c we show the squared coefficients $|C_i(t)|^2$ of the quantum state following the optimal trajectory. From Fig. 9c we show that the final state with unit concurrence is not Bell state, because all coefficients are not zero.

## Conclusions

In this paper we investigate the optimal control problem in a symmetrically coupled two-qubit system with bounded amplitude. By optimizing over the family of bang-off controls, the problems of quantum state preparation and entanglement creation are studied. Given the initial states, the cost functions—fidelity and concurrence—are optimized for various duration. By studying the difference of best cost function obtained with different types of control field, optimal controls and critical time points are determined more accurately than the previous work. In addition, we employ the CRAB method as a comparison, and its performance, in general, is not as good as our method, concerning the best fidelity obtained.

For the quantum state preparation problem, we have shown that for duration below a threshold value, the optimal control fields are bang-off, whereas this is not true for duration larger than that threshold value. However, the bang-off control approximates the optimal control very well concerning the best fidelity obtained. Furthermore, we estimate the quantum speed limit and time-optimal control field using the bang-off control. The QSL obtained is equal to that obtained with GRAPE, but the temporal shape of bang-off control is simple.

For the entanglement creation problem, we start from the product state and maximize the concurrence using bang-off controls for different durations. Two critical time points are obtained. For duration in the overconstrained phase, the optimal control is simple: the control field takes either the maximal value or the minimal. As duration increases, the optimal control is of bang-off type with $N_s=3$. The minimal duration to reach the unit concurrence is estimated, and time-optimal control is obtained.

Considering the fact that in quantum systems the optimal control is sometimes not bang-bang anymore, bang-off control serves as a good approximation of the optimal control, because of its simplicity. The bang-off control can be applied in the quantum system with larger size, e.g., the few-body and many-body system. And it is interesting to compare the bang-off control with other control protocols for these quantum systems.

## Data Availability

The datasets used and/or analysed during the current study available from the corresponding author on reasonable request.
